# Racial Disparities in Access to High-Volume Mitral Valve Transcatheter Edge-to-Edge Repair Centers

**DOI:** 10.1016/j.jscai.2022.100398

**Published:** 2022-07-13

**Authors:** Diala Steitieh, Alyssa Zaidi, Shirley Xu, Jim W. Cheung, Dmitriy N. Feldman, Mark Reisman, Sonal Mallya, Tracy K. Paul, Harsimran S. Singh, Geoffrey Bergman, Krista Vadaketh, Mostafa Naguib, Robert M. Minutello, Shing Chiu Wong, Nivee P. Amin, Luke K. Kim

**Affiliations:** a Division of Cardiology, Department of Medicine, Weill Cornell Medical College, NewYork-Presbyterian Hospital, New York, New York; b Department of Medicine, Weill Cornell Medical College, NewYork-Presbyterian Hospital, New York, New York; c Fordham University, Bronx, New York; d Weill Cornell Cardiovascular Outcomes Research Group (CORG), Division of Cardiology, Department of Medicine, Weill Cornell Medical College, NewYork-Presbyterian Hospital, New York, New York; e Department of Medicine, Morristown Medical Center, Morristown, New Jersey; f Division of Cardiology, Department of Medicine, Weill Cornell Medical College, NewYork-Presbyterian Hospital Queens, New York, New York

**Keywords:** disparities, edge-to-edge, minorities, transcatheter edge-to-edge repair, transcatheter mitral valve repair

## Abstract

**Background:**

Severe mitral regurgitation is a progressive disease associated with high morbidity and mortality, and frequent readmissions for heart failure. Surgical mitral valve repair or replacement has been the gold-standard treatment; however, advances in transcatheter edge-to-edge repair (TEER) have provided alternatives for high-risk surgical patients. There are no data on racial disparities in access to high-volume TEER centers.

**Methods:**

Data on TEER hospitalizations from New York, New Jersey, Maryland, North Carolina, Washington, Colorado, Arizona, and Florida were analyzed using the State Inpatient Databases for 2016. The baseline characteristics of patients who underwent TEER at high- (≥25 procedures per year) and low-volume centers were identified. The association between race and the likelihood of undergoing TEER at high-volume centers was assessed. The secondary outcomes were mortality and the frequency of home discharges.

**Results:**

Of 1567 patients included in the analysis, 1129 underwent TEER at high-volume centers. Patients treated at high-volume centers had a higher prevalence of chronic kidney disease and congestive heart failure. Black and Hispanic patients were 59% (adjusted odds ratio [OR], 0.41; *P* < .001) and 51% (adjusted OR, 0.49; *P* < .001) less likely to undergo TEER at high-volume centers, respectively, compared with White patients. Hispanic patients were 3 times more likely to die during index admission than White patients (adjusted OR, 3.32; *P* = .027). There was geographic clustering of TEER centers, and a higher ratio of White patients to minority patients in zip codes with high-volume TEER centers.

**Conclusions:**

Racial minorities patients, particularly Black and Hispanic patients, are less likely to undergo TEER at high-volume centers. Hispanic patients experience higher rates of in-hospital mortality after TEER than White patients.

## Highlights

• Over 80% of patients undergoing transcatheter edge-to-edge repair (TEER) are White.
• Racial minorities undergoing TEER have more comorbidities than White patients.
• Black and Hispanic patients are less likely to access high-volume transcatheter edge-to-edge repair centers.
• Hispanic patients are 3 times more likely to die during admission for TEER.

## Introduction

Mitral regurgitation (MR) is the most frequent valvular disease in the United States, affecting >10% of people aged ≥75 years, with increasing prevalence.^1^^,^^2^ Severe MR is associated with a significantly elevated risk of mortality and readmissions for heart failure.^3^ Surgical valvular repair or replacement remains the standard of care for these patients^4^; however, advances in transcatheter edge-to-edge repair (TEER) have provided alternatives for high-risk surgical patients. The advent of TEER has been supported by the COAPT trial (Cardiovascular Outcomes Assessment of the MitraClip Percutaneous Therapy for Heart Failure Patients with Functional Mitral Regurgitation), which demonstrated a 32.1% absolute reduction in hospitalization for heart failure at 2 years and a 16% absolute reduction in all-cause mortality for those with moderate-to-severe or severe functional MR.^5^ Endovascular Valve Edge-to-Edge REpair STudy then demonstrated a similar efficacy of TEER as that of surgery, but with superior safety with TEER.^6^ As a result, TEER is now the standard therapy for patients who are at a prohibitive risk of surgical repair.

Previous studies have shown a racial disparity in access to cardiac procedures, including coronary artery bypass graft,^7^ surgical mitral valve repair or replacement,^8^ and percutaneous coronary interventions,^7^ and advanced structural heart disease interventions, such as transcatheter aortic valve replacement (TAVR) and left atrial appendage occlusion.^9^^,^^10^ However, it is not known whether there are racial disparities in access to experienced TEER centers. High-volume centers are associated with improved outcomes and lower rates of readmission,^11^ and high-volume operators have a higher likelihood of procedural success.^12^ Therefore, equal access to these centers is imperative, especially for complex cases. Using the State Inpatient Databases (SID), we aimed to investigate the racial disparities in access to high-volume TEER centers and the difference in the outcomes of TEER.

## Methods

### Data source and study population

A retrospective analysis was conducted using the Healthcare Cost and Utilization Project’s (HCUP) SID for 2016. SID represents roughly 95% of all hospital discharges in the United States and allows multistate comparisons of patient and hospital characteristics. Data from the following states were included in our analysis: New York, New Jersey, Maryland, North Carolina, Washington, Colorado, Arizona, and Florida. These states were chosen to represent various demographic regions across the country.

Each patient record in SID contains information on the patient’s diagnoses and procedures performed during hospitalization, based on International Classification of Diseases, Tenth Revision–Clinical Modification (ICD-10-CM) codes. Institutional review board approval and informed consent were not required for the study because all data collected were derived from a publicly available, deidentified administrative database.

**Figure. 1:**
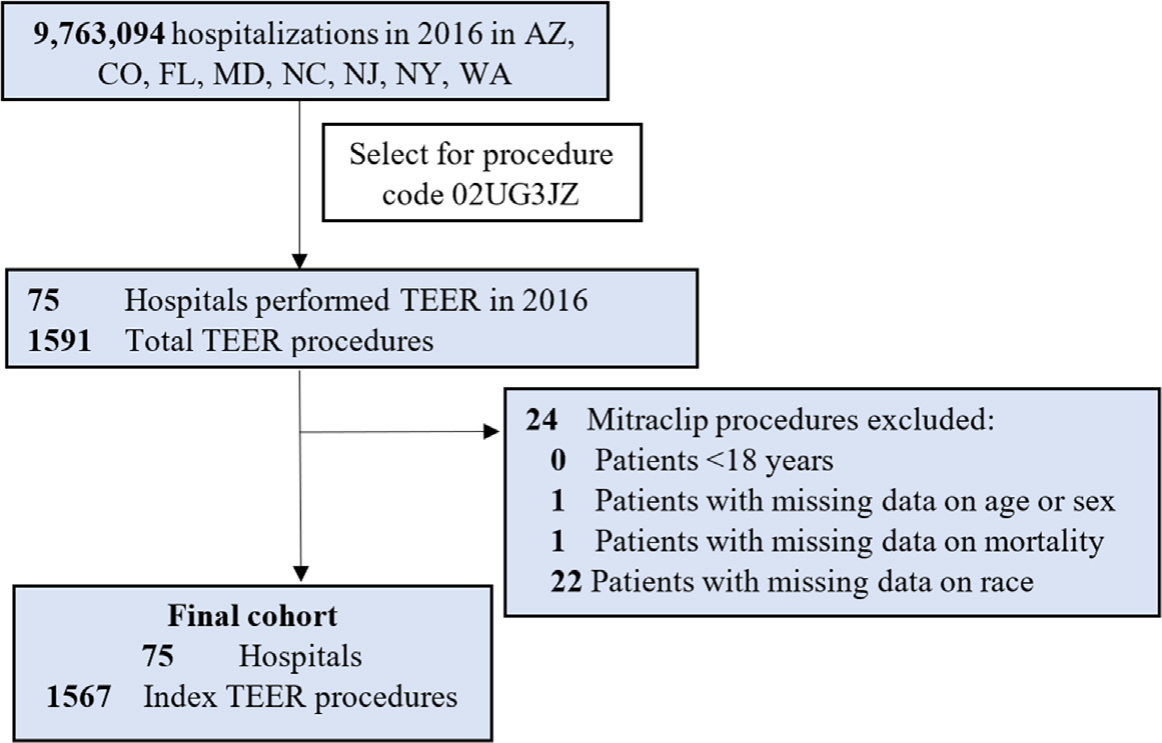
**Study cohort.** The inclusion and exclusion criteria are noted, with the final cohort including 75 hospitals and 1567 transcatheter edge-to-edge repair procedures. AZ, Arizona; CO, Colorado; FL, Florida; MD, Maryland; NC, North Carolina.; NJ, New Jersey; NY, New York; WA, Washington; TEER, transcatheter edge-to-edge repair.

Using ICD-10-CM codes, we identified hospitalizations between January 2016 through December 2016 based on the procedure code for TEER (02UG3JZ). Patients were excluded if they were <18 years of age or had missing information on mortality, race, or sex (Figure 1).

### Study variables

The patient-specific variables included for the analysis were age, sex, comorbidities, race, median household income (by quartile, determined by HCUP as the average income for a specific zip code), weekend admission, elective admission, length of stay, total mean cost of treatment, mortality, and home discharge. The minority patients included in the study were mainly Black and Hispanic patients, and the other minorities were Asian, Pacific Islander, and Native American.

Past medical history of smoking, hypertension, diabetes mellitus (DM), dyslipidemia, myocardial infarction, congestive heart failure (CHF), peripheral vascular disease, chronic kidney disease (CKD), atrial fibrillation, coagulopathy, collagen vascular disease, electrolyte abnormalities, obesity, and deep vein thrombosis or pulmonary embolism was identified using ICD-10-CM codes (Supplemental Table S1). The hospital-specific HCUP code was matched with the corresponding zip code for each TEER center.

### Study end points

The primary outcome was access to high-volume TEER centers, stratified by race. There has been debate on whether an annual volume metric for TEER is associated with improved safety outcomes. Previous studies have shown that centers that perform >25 MitraClip procedures a year have lower rates of surgical bailout and cardiovascular readmission as well as shorter hospital stays.^13^^,^^14^ Therefore, the high-volume centers in our study were defined as centers that performed ≥25 TEERs per year.

The secondary outcomes were mortality during index hospitalization and the frequency of home discharges. We also studied the geographic locations of the TEER centers, as well as patient zip codes, stratified by race, to further analyze the racial disparity. In order to highlight the differences in access to high-volume TEER centers, the ratio of White patients to minority patients who underwent TEER at each TEER center was calculated.

### Statistical analysis

All analyses were performed using STATA, version 15.1 (Statacorp). For descriptive analyses, we compared the baseline characteristics of the patients between the cohorts and then stratified by race. For comparison, the Pearson χ^2^ test was used for categorical variables, and survey-specific linear regression was used for continuous variables. An alpha error rate of *P* ≤.05 was considered statistically significant. To assess the association of race with mortality and disposition, a multivariate logistic regression analysis was performed. Univariate and multivariate logistic regression analyses were performed to assess the effect of race on the likelihood of undergoing TEER at high-volume centers.

## Results

### Study population and baseline characteristics

The 2016 SID data sets included a total number of 1164 hospitals (Supplemental Table S2). Of the 9,763,094 discharge records reviewed, 1591 hospital discharges involved TEER. After excluding 24 cases because of missing data, 1567 patients were included in the analysis (47% women and 53% men). In this cohort, 72% of the patients underwent TEER at high-volume centers. Table 1 shows the baseline characteristics of patients at low- and high-volume centers. There was no significant difference in age, sex, and the frequency of hypertension, DM, dyslipidemia, and obesity between these 2 cohorts. However, CHF (69.1% vs 61.2%, *P* < .001) and CKD (41.5% vs 36.1%, *P* = .05) were more common in patients who underwent TEER at high-volume centers. The racial minorities represented a small portion of the total population that underwent TEER (Table 2; 88.8% of the patients were White compared with 5.9% Black and 5.3% Hispanic patients). Among those admitted for TEER, Blacks and Hispanics were generally younger in age and more likely to be women. When the difference in comorbidities among the races was assessed, we found that the minority patients had a higher prevalence of comorbidities such as DM, CKD, and obesity (although there was also a difference in the prevalence of these comorbidities among the 3 racial minority subgroups). Additionally, the minority patients were significantly less likely to undergo TEER during elective admissions (70.1% of White patients underwent elective admissions compared with only 56.5% of Black patients and 57.8% of Hispanic patients, *P* < .001).

**Table 1:** Baseline characteristics of patients admitted for transcatheter edge-to-edge repair by hospital volume.

Characteristic	Overall N = 1567	Low volume n = 438	High volume n = 1129 (72.1%)	*P* value^a^
Age, y (mean)	78.9	78.5	79.2	.44^b^
18-45	17 (1.1)	4 (0.9)	13 (1.2)	.62
46-65	141 (9.0)	44 (10.1)	97 (8.6)	
>65	1409 (89.9)	390 (89.0)	1019 (90.3)	
Female	735 (46.9)	214 (48.9)	521 (46.2)	.33
Patient characteristics				
Smoking	605 (38.6)	178 (40.6)	427 (37.8)	.30
Hypertension	631 (40.3)	193 (44.1)	438 (38.8)	.06
Diabetes	405 (26.2)	116 (26.5)	289 (25.6)	.72
Dyslipidemia	782 (49.9)	235 (53.7)	547 (48.5)	.07
Prior MI	243 (15.5)	77 (17.6)	166 (14.7)	.16
CHF	1048 (66.9)	268 (61.2)	780 (69.1)	<.001
PVD	143 (9.13)	40 (9.1)	103 (9.1)	.10
CKD	627 (40.0)	158 (36.1)	469 (41.5)	.05
Atrial fibrillation	971 (62.2)	280 (63.9)	691 (61.2)	.32
Electrolyte abnormalities	254 (16.2)	63 (14.4)	191 (16.9)	.22
Obesity	154 (9.8)	42 (9.6)	112 (9.9)	.84
Race				
White	1314 (83.9)	340 (77.6)	974 (86.3)	<.001
Black	92 (5.9)	40 (9.1)	52 (4.6)	
Hispanic	83 (5.3)	40 (9.1)	43 (3.8)	
Other	78 (5.0)	18 (4.1)	60 (5.3)	
Median household income, percentile for patient zip code				
First quartile	312 (20.4)	91 (21.1)	221 (20.1)	.88
Second quartile	354 (23.1)	95 (22.4)	259 (23.5)	
Third quartile	411 (26.8)	113 (26.2)	298 (27.0)	
Fourth quartile	456 (29.8)	132 (30.6)	324 (29.4)	
Admission and hospital characteristics				
Weekend admission	62 (4)	16 (3.7)	46 (4.1)	.70
Elective admission	1060 (67.6)	326 (74.4)	734 (65.0)	<.001
Length of stay, d				
1-3	1027 (65.5)	310 (70.8)	717 (63.5)	.13^b^
4-5	172 (11.0)	42 (9.6)	130 (11.5)	
>6	368 (23.5)	86 (19.6)	282 (25.0)	
Total cost, $ (mean)	232,299.4	204,022.1	243,269.7	<.001^b^

Values are presented as n (%), unless otherwise indicated.

CHF, congestive heart failure; CKD, chronic kidney disease; MI, myocardial infarction; PVD, peripheral vascular disease.

a The Rao-Scott χ^2^ test was used for all statistical tests in this table, unless stated otherwise.

b Linear regression was performed.

**Table 2:** Baseline characteristics of patients admitted for transcatheter edge-to-edge repair, stratified by race.

Characteristic	Overall N = 1567	White n = 1314	Black n = 92	Hispanic n = 83	Other n = 78	*P* value^a^
Age, y (mean)	78.9	79.9	70.5	75.4	76.9	<.001^b^
18-45	17 (1.1)	7 (0.5)	6 (6.5)	3 (3.6)	1 (1.3)	
46-65	141 (9.0)	96 (7.3)	23 (25.0)	12 (14.5)	10 (12.8)	<.001
>65	1409 (89.9)	1211 (92.2)	63 (68.5)	68 (81.9)	67 (85.9)	
Female	735 (46.9)	596 (45.4)	55 (59.8)	46 (55.4)	38 (48.7)	.02
Patient characteristics						
Smoking	605 (38.6)	524 (39.9)	29 (31.5)	26 (31.3)	26 (33.3)	.13
Hypertension	631 (40.3)	548 (41.7)	19 (20.7)	34 (41.0)	30 (38.5)	<.001
Diabetes	405 (26.2)	304 (23.1)	45 (48.9)	36 (43.4)	20 (25.6)	<.001
Dyslipidemia	782 (49.9)	666 (50.7)	38 (41.3)	44 (53.0)	34 (43.6)	.20
Prior MI	243 (15.5)	192 (14.6)	14 (15.2)	23 (27.7)	14 (18.0)	.01
CHF	1048 (66.9)	857 (65.2)	74 (80.4)	55 (66.3)	62 (79.5)	<.001
CKD	627 (40.0)	488 (37.1)	66 (71.7)	39 (47.0)	34 (43.6)	<.001
Atrial fibrillation	971 (62.2)	840 (63.9)	51 (55.4)	39 (47)	41 (52.6)	<.001
Electrolyte abnormalities	254 (16.2)	202 (15.4)	23 (25)	16 (19.3)	13 (16.7)	.09
Obesity	154 (9.8)	120 (9.1)	20 (21.7)	13 (15.7)	1 (1.3)	<.001
Median household income, percentile for patient zip code						
First quartile	312 (20.4)	225 (17.4)	40 (45.5)	26 (32.9)	21 (28.0)	<.001
Second quartile	354 (23.1)	296 (22.9)	29 (33.0)	14 (17.7)	15 (20.0)	
Third quartile	411 (26.8)	353 (27.3)	12 (13.6)	25 (31.7)	21 (28.0)	
Fourth quartile	456 (29.8)	417 (32.3)	7 (8.0)	14 (17.7)	18 (24.0)	
Admission and hospital characteristics						
Elective admission	1060 (67.6)	921 (70.1)	52 (56.5)	48 (57.8)	39 (50.0)	<.001
Length of stay, d						
1-3	1027 (65.5)	899 (68.4)	45 (48.9)	50 (60.2)	33 (42.3)	
4-5	172 (11.0)	137 (10.4)	15 (16.3)	7 (8.4)	13 (16.7)	<.001^b^
>6	368 (23.5)	278 (21.2)	32 (34.78)	26 (31.3)	32 (41.0)	
Total cost, $ (mean)	232,299.4	229,249.8	234,346.2	266,756.2	244,592.6	<.001^b^

Values are presented as n (%), unless otherwise indicated.

CHF, congestive heart failure; CKD, chronic kidney disease; MI, myocardial infarction.

a The Rao-Scott χ^2^ test was used for all statistical tests in this table, unless stated otherwise.

b Linear regression was performed.

### Racial disparity in access to high-volume TEER centers

A multivariate analysis was performed to assess the racial disparities in access to high-volume TAVR centers (Central Illustration). When all the minorities were assessed, there was no difference in the likelihood of accessing high-volume TEER centers. However, Black and Hispanic patients were considerably less likely to undergo TEER at high-volume centers than White patients when each race was analyzed separately. Specifically, Black and Hispanic patients had 59% lower chances (adjusted odds ratio [OR], 0.41; 95% CI, 0.27-0.62; *P* < .001) and 51% lower chances (adjusted OR, 0.49; 95% CI, 0.31-0.77; *P* < .001) of undergoing TEER at high-volume centers, respectively.

**Central Illustration:**
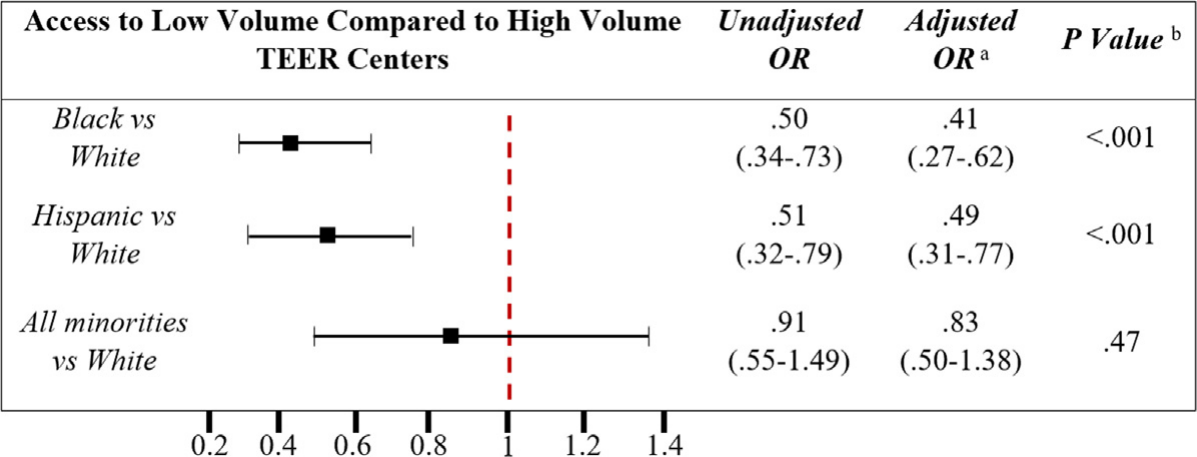
**Unadjusted and adjusted association between race and the likelihood of undergoing transcatheter edge-to-edge repair at high-volume centers.** OR, odds ratio; TEER, transcatheter edge-to-edge repair. ^a^The models were adjusted for significant variables: median household income, sex, history of smoking, hypertension, diabetes, dyslipidemia, prior myocardial infarction, congestive heart failure, peripheral vascular disease, pulmonary hypertension, obesity, atrial fibrillation, coagulopathy, collagen vascular disease, and electrolyte abnormalities. ^b^The Rao-Scott χ^2^ test was used for all statistical tests in this table.

Hispanic patients were 3 times more likely to die during their index admission after TEER than White patients (Table 3; adjusted OR, 3.32; 95% CI, 1.15-9.63; *P* = .03). This discrepancy was not observed in the other racial minorities. The proportion of home discharges, however, was not different among all the races.

**Table 3:** Racial differences in mortality and disposition after transcatheter edge-to-edge repair.

In-hospital outcomes	Race	Unadjusted OR (95% CI)	Adjusted OR (95% CI)^a^	*P* value^b^
Mortality	White	Reference		
	Black	0	0	
	Hispanic	3.98 (1.46-10.79)	3.32 (1.15-9.63)	.03
	Black or Hispanic	1.47 (0.55-3.92)	1.2 (0.42-3.46)	.73
	All minorities	1.29 (0.52-3.21)	1.07 (0.41-2.83)	.89
Home discharge	White	Reference		
	Black	0.74 (0.50-1.09)	0.93 (0.62-1.40)	.74
	Hispanic	1.03 (0.65-1.63)	1.10 (0.68-1.79)	.6
	Black or Hispanic	0.85 (0.62-1.15)	0.996 (0.72-1.38)	.98
	All minorities	0.74 (0.57-0.96)	0.82 (0.62-1.09)	.17

OR, odds ratio.

a The models were adjusted for significant variables: median household income, sex, history of smoking, hypertension, diabetes, dyslipidemia, prior myocardial infarction, congestive heart failure, peripheral vascular disease, pulmonary hypertension, obesity, atrial fibrillation, coagulopathy, collagen vascular disease, and electrolyte abnormalities.

b The Rao-Scott χ^2^ test was used for all statistical tests in this table.

### Geographic differences in high-volume TEER centers

Supplemental Table S3 outlines all TEER hospital zip codes as well as the number of minority and White patients who underwent TEER in each zip code. The ratio of White patients to minority patients who underwent TEER was especially high in several zip codes, and these zip codes, seen in Supplemental Table S3, correlated with the locations of high-volume centers. There was no interaction between socioeconomic status (median household income) and race (*P* value for this interaction was .83).

## Discussion

Our analysis of SID is the first to examine the racial disparities in access to high-volume TEER centers. There are several important findings in our study. The racial minority patients, specifically Black and Hispanic patients, had lesser access to high-volume TEER centers than White patients. After TEER, Hispanic patients experienced higher mortality than patients from other races. Finally, although there was geographic clustering of TEER centers observed across the United States, the ratio of White patients to minority patients was especially high in zip codes with high-volume centers.

Consistent with the findings of previous studies,^10^ we found that the majority of patients (>80%) who underwent TEER were White and that Black and Hispanic patients represented a very small minority of the total patient population that underwent TEER (5.9% and 5.3%, respectively). This racial disparity has also been shown for other structural heart interventions, such as TAVR.^15^ Although there is limited information on the racial difference in the prevalence of MR in the general population, the Atherosclerosis Risk In Communities study showed no association between race and the prevalence of moderate or severe MR in patients hospitalized for heart failure.^16^ Therefore, factors that contribute to these disparities in access to TEER may instead be differences in socioeconomic factors^9^^,^^17^ and subsequently lower the rates of preventive health care in ethnic minorities, which may ultimately result in fewer referrals for TEER.^9^ This discrepancy may also be explained by a lack of readily accessible advanced structural heart disease centers and differences in the willingness to undergo invasive or surgical procedures in racial minority patients.^18^ Previous studies have demonstrated a racial difference in patient preference for undergoing surgical or invasive procedures, such as coronary artery bypass graft, surgical aortic valve replacement, and TAVR.^19^, ^20^, ^21^, ^22^ The measure of familiarity with the procedure was the most significant predictor of the likelihood of undergoing invasive procedures. The Association of Black Cardiologists examined the barriers in accepting invasive treatment for valvular disease^23^ and found that unfamiliarity with the procedure is one of the largest contributing factors. The possible solutions to this dilemma include improved patient outreach and education as well as education of family members and caregivers who are more likely to be involved in the decision-making process of minority patients.^24^

Our study confirmed previous findings that patients who undergo TEER at high-volume centers are sicker, with a higher prevalence of CHF and CKD. Despite more complex patient populations undergoing TEER at high-volume centers, our study demonstrated that patients treated at high-volume centers have a similar length of stay, disposition, and mortality compared with those treated at low-volume centers. Although our study was not designed to assess procedural success, Chhatriwalla et al^25^ previously demonstrated that the successful elimination of MR after TEER (defined as ≤1 + MR after procedure) is more frequently achieved at experienced centers. This is likely related to the complex nature of TEER, where experience in device delivery by the interventional cardiologist and guidance by a structural echocardiographer are essential.^26^ In addition, a previous study demonstrated a clear association between higher volumes of TEER and significantly fewer procedural complications.^27^ This is especially important, given the higher prevalence of “sicker” patients among the racial minorities in our study. A follow-up study of the Endovascular Valve Edge-to-Edge REpair STudy cohort showed that patients with a higher burden of comorbidities experience higher mortality rates after index hospitalization after TEER.^28^ Therefore, access to high-volume centers is critical for improved TEER outcomes in high-risk populations, including racial minorities with complex mitral anatomy.

In contrast to previous studies,^10^ we demonstrated that certain racial minority groups experience higher mortality after TEER. In our study, Hispanic patients were approximately 3 times more likely to die during their index admission than White patients. Urgent TEER has been associated with worse outcomes,^29^^,^^30^ and several studies have demonstrated higher rates of admissions for urgent TEER among Hispanic patients. In our study, 42.2% of Hispanic patients presented for urgent TEER compared with 29.9% of White patients. The higher prevalence of admissions for urgent TEER among Hispanic patients may be a result of the increased prevalence of comorbidities,^31^ delayed diagnosis, and suboptimal treatment because of poor follow-up, which may have led to a more severe disease process at the time of presentation.^29^ Nevertheless, it is important to recognize that mortality after TEER is quite rare, and therefore, focus on major adverse cardiovascular events for safety outcomes should be considered for further investigations.

The observed geographic differences in access to TEER show that the majority of TEER centers are within clusters of densely populated cities. The growth of TEER centers in these areas is similar to that observed for high-volume centers that perform TAVR^32^ and percutaneous coronary interventions.^33^ Although improving the distribution of TEER centers may help more patients access them, it is unlikely to solely resolve the disparity in access to high-volume centers. Instead, we recommend focusing on adequate follow-up of patients with MR, with access to quality outpatient cardiovascular care, improved patient education, and advocacy for TEER, especially in racial minority patients.

### Limitations

The results of this study should be interpreted in the context of several limitations. First, although we were able to use ICD-10-CM codes to analyze data, miscoded and missing information can occur in large administrative data sets such as SID. However, HCUP quality control procedures are regularly performed to confirm that the SID data values are valid, consistent, and reliable.^34^ Secondly, SID only includes data that can be encoded by ICD-10-CM codes and, therefore, cannot include detailed information, such as mitral valve anatomy or the severity of residual MR after TEER, for the assessment of the success of TEER at high- vs low-volume centers. Third, SID does not contain granular patient-level data, such as the use of medications, baseline ejection fraction, and laboratory values. Fourth, although we studied a variety of states across the country, this does not guarantee that the same patterns in access to high-volume TEER centers are present in every state. Fifth, the number of minority patients was considerably low (92 Black and 82 Hispanic patients), which might have introduced a bias in the results. Finally, SID provides retrospective data, and therefore, the long-term outcomes of TEER could not be assessed.

## Conclusion

Our study highlights important racial differences in access to high-volume TEER centers and the outcomes of TEER. Black and Hispanic patients were less likely to undergo TEER at high-volume centers, and Hispanic patients experienced a higher risk of mortality after TEER than White patients. There is geographic clustering of TEER centers, and zip codes with high-volume centers had a high ratio of White patients to minority patients. This study highlights the need for further race-based analyses in order to address this disparity in access to TEER.

## Declaration of competing interest

Dr Kim has received fellowship grant support from Abbott and Medtronic. Drs Steitieh, Zaidi, Xu, Cheung, Feldman, Reisman, Mallya, Paul, Singh, Bergman, Vadaketh, Naguib, Minutello, and Wong and Author Amin reported no financial interests.

## Funding sources

This work was supported by grants from the 10.13039/100010617Michael Wolk Heart Foundation, New York, NY, United States; the New York Cardiac Center, Inc, New York, NY; and the New York 10.13039/100007273Weill Cornell Medical Center Alumni Council, New York, NY, United States. The Michael Wolk Heart Foundation, the New York Cardiac Center, Inc, and the New York Weill Cornell Medical Center Alumni Council had no role in the design and conduct of the study, in the collection, analysis, and interpretation of the data, or in the preparation, review, or approval of the manuscript.

## Ethics statement

The research reported adheres to the ethical guidelines.
