# Supplementary material for: Racial Disparities in Access to High-Volume Mitral Valve Transcatheter Edge-to-Edge Repair Centers
Source: J Soc Cardiovasc Angiogr Interv. 2022 Jul 13;1(5):100398. doi: 10.1016/j.jscai.2022.100398 (PMC11308692; doi:10.1016/j.jscai.2022.100398)
Supplement: Supplementary Tables 1-3 [file mmc1.docx]

Supplemental table I: ICD-10-CM codes for comorbidities

| **Comorbidity** | **ICD-10-CM code** |
| --- | --- |
| Smoking | F172*, Z87891 |
| Hypertension | I10 |
| Diabetes | E10*, E11*, |
| Dyslipidemia | E785 |
| Prior myocardial infarction | I252 |
| Congestive heart failure | I501, I5020*, I5021, I5022, I5023, I5030*, I5031, I5032, I5033, I5040*, I5041, I5042, I5043, I5090* |
| Peripheral vascular disease | I702*, I703*, I704*, I705*, I706*, I707*, I739 |
| Chronic kidney disease | N181, N182, N183*, N184, N185, N186, N189 |
| Atrial fibrillation or atrial flutter | I480, I481, I482, I483, I484, I4891* , I4892* |
| Coagulopathy | D65, D66, D67, D68.0, D68.1, D68.2, D68.3*, D68.4 |
| Collagen vascular disease | M30*, M31*, M32*, M33*, M34*, M35*, M36* |
| Electrolyte abnormalities | E87* |
| Obesity | Z6830, Z6831, Z6832, Z6833, Z6834, Z6835, Z6836, Z6837, Z6838, Z6839, Z6840, Z6841, Z6842, Z6843, Z6844, Z6845, E6601, E6609, E663, E668, E669 |
| History of thromboembolism | I824*, I824Y*, I824Z*, I825*, I825Y* |

Supplemental table II: Total Number of Hospitals Per State

| **State** | **Total Discharges** | **Hospitals** |
| --- | --- | --- |
| **AZ** | 735,890 | 115 |
| **CO** | 480,573 | 87 |
| **FL** | 2,837,863 | 299 |
| **MD** | 622,815 | 52 |
| **NC** | 1,110,146 | 128 |
| **NJ** | 979,099 | 114 |
| **NY** | 2,347,084 | 212 |
| **WA** | 649,624 | 157 |
| **Total** | 9,763,094 | 1164 |

Supplemental Table III: Distribution of TMVR Centers by Zipcodes, and Patient Access By Race

| **State** | **Zipcode** | **Volume** | **Minorities: Black & Hispanic Patients** | **White Patients** | **Ratio of W:M** |
| --- | --- | --- | --- | --- | --- |
| AZ | 85006 | High | 10 | 64 | 6.4 |
|  | 85260 | High | 0 | 26 | 26 |
|  | 85308 | High | 3 | 46 | 15.3 |
|  | 85712 | High | 6 | 23 | 3.8 |
|  | 85054 | Low | 2 | 12 | 6 |
|  | 85013 | Low | 9 | 13 | 1.4 |
|  | 85719 | Low | 0 | 7 | 7 |
|  | 85745 | Low | 2 | 4 | 2 |
| CO | 80045 | High | 7 | 32 | 4.6 |
|  | 80631 | Low | 0 | 9 | 9 |
|  | 80907 | Low | 2 | 9 | 4.5 |
|  | 80012 | Low | 0 | 4 | 4 |
|  | 80538 | Low | 1 | 17 | 17 |
| FL | 34208 | High | 0 | 45 | 45 |
|  | 33756 | High | 1 | 43 | 43 |
|  | 32501 | High | 2 | 23 | 11.5 |
|  | 33410 | High | 2 | 26 | 13 |
|  | 32207 | High | 4 | 24 | 6 |
|  | 32308 | High | 4 | 37 | 9.3 |
|  | 32803 | High | 15 | 43 | 2.9 |
|  | 33462 | High | 7 | 45 | 6.4 |
|  | 33484 | High | 3 | 41 | 13.7 |
|  | 32204 | Low | 5 | 2 | 0.4 |
|  | 33021 | Low | 3 | 4 | 1.3 |
|  | 33176 | Low | 17 | 11 | 0.7 |
|  | 33901 | Low | 0 | 8 | 8 |
|  | 33140 | Low | 5 | 4 | 0.8 |
|  | 34741 | Low | 1 | 0 | 0.1 |
|  | 33709 | Low | 0 | 1 | 1 |
|  | 33063 | Low | 1 | 0 | 0.1 |
|  | 33016 | Low | 3 | 0 | 0.3 |
|  | 33486 | Low | 0 | 2 | 2 |
|  | 32073 | Low | 0 | 1 | 1 |
|  | 32401 | Low | 0 | 1 | 1 |
|  | 33606 | Low | 5 | 8 | 1.6 |
|  | 32806 | Low | 2 | 2 | 1 |
|  | 32224 | Low | 1 | 8 | 8 |
|  | N/A | Low |  |  |  |
|  | 33607 | Low | 0 | 4 | 4 |
|  | 34239 | Low | 1 | 11 | 11 |
| MD | 20814 | Low | 0 | 1 | 1 |
|  | 21201 | Low | 2 | 10 | 5 |
| NC | 28204 | High | 6 | 20 | 3.33 |
|  | 28203 | High | 6 | 32 | 5.33 |
|  | 27103 | Low | 1 | 11 | 11 |
|  | 27157 | Low | 3 | 13 | 4.33 |
|  | 27514 | Low | 4 | 13 | 3.25 |
|  | 27834 | Low | 1 | 0 | 0.1 |
|  | 27607 | Low | 2 | 11 | 5.5 |
|  | 27401 | Low | 0 | 1 | 1 |
|  | 27705 | Low | 2 | 12 | 6 |
|  | 27610 | Low | 0 | 2 | 2 |
| NJ | 07960 | High | 3 | 29 | 9.7 |
|  | 07450 | Low | 0 | 9 | 9 |
|  | 07112 | Low | 3 | 7 | 2.3 |
|  | 07601 | Low | 0 | 2 | 2 |
|  | 07754 | Low | 0 | 1 | 1 |
| NY | 14203 | High | 5 | 28 | 5.6 |
|  | 10595 | High | 6 | 30 | 5 |
|  | 10016 | High | 16 | 47 | 2.9 |
|  | 10065 | High | 25 | 23 | 0.9 |
|  | 11706 | High | 3 | 17 | 5.7 |
|  | 11030 | Low | 7 | 14 | 2 |
|  | 13203 | Low | 2 | 6 | 3 |
|  | 10029 | Low | 4 | 9 | 2.3 |
|  | 11576 | Low | 2 | 15 | 7.5 |
|  | 10075 | Low | 4 | 4 | 1 |
|  | 11706 | Low | 3 | 17 | 5.7 |
|  | 10467 | Low | 12 | 3 | 0.3 |
| WA | 99204 | High | 2 | 17 | 8.5 |
|  | 98122 | High | 4 | 29 | 7.3 |
|  | 98195 | High | 6 | 50 | 8.3 |
|  | 98004 | Low | 0 | 5 | 5 |
|  | 98405 | Low | 1 | 6 | 6 |
